# Trends of Medication Usage and Associated Outcomes for Taiwanese Patients with Inflammatory Bowel Disease from 2001 to 2015

**DOI:** 10.3390/jcm7110394

**Published:** 2018-10-27

**Authors:** Meng-Tzu Weng, Chien-Chih Tung, Yuan-Ting Chang, Yew-Loong Leong, Yu-Ting Wang, Jau-Min Wong, Shu-Chen Wei

**Affiliations:** 1 Departments of Internal Medicine, National Taiwan University Hospital, Taipei 100, Taiwan; wengmengtzu@gmail.com (M.-T.W.); jmwong@ntu.edu.tw (J.-M.W.); 2 Department of Internal Medicine, Far Eastern Memorial Hospital, New Taipei 220, Taiwan; 3 Department of Chemical Engineering & Materials Science, Yuan-Ze University, Taoyuan 320, Taiwan; 4 Department of Integrated Diagnostics & Therapeutics, National Taiwan University Hospital, Taipei 100, Taiwan; cnicemike@yahoo.com.tw; 5 Health Data Research Center, National Taiwan University, Taipei 106, Taiwan; glot1024@gmail.com; 6 Department of Internal Medicine, West Garden Hospital, Taipei 108, Taiwan; leong@westgarden.com.tw; 7 Departments of Medical Research, National Taiwan University Hospital, Taipei 100, Taiwan; wangyt@ntuh.gov.tw; 8 Inflammatory Bowel Disease Clinical and Study Integrated Center, National Taiwan University Hospital, Taipei 100, Taiwan

**Keywords:** inflammatory bowel disease, 5-aminosalicylic acid, corticosteroids, thiopurine, anti-tumor necrosis factor-α agents

## Abstract

Background: No nationwide, long-term follow-up study has assessed medication-associated outcomes for Asian patients with inflammatory bowel disease (IBD). This study examined medication-associated outcomes for Taiwanese patients with IBD. Methods: In this nationwide cohort study, 3806 patients who had received catastrophic illness registration for IBD from 2001 to 2015 were enrolled. Results: A higher accumulated dosage of 5-aminosalicylic acid (5-ASA) was associated with decreased risks of hospitalization (hazard ratio (HR) = 0.6) and operation (HR = 0.5). Thiopurine was associated with increased risks of hospitalization (HR = 2.1 in the high-dosage group) and tuberculosis (TB; HR = 3.6) reactivation but not with operation risk. A higher accumulated dosage of anti-TNF-α agents was associated with increased risks of hospitalization (HR = 3.3), operation (HR = 2.9), hepatitis B (HR = 4.3), and TB (HR = 5.1) reactivation. Corticosteroids were associated with increased risks of hospitalization (HR = 3.5 in the high-dosage group), risk of operation, hepatitis B (HR = 2.8) and TB (HR = 2.8) reactivation. Conclusions: 5-ASA usage is associated with decreased risks of hospitalization and operation for patients with IBD, whereas thiopurine, corticosteroids, and anti-TNF-α agents are associated with increased risks of hospitalization and hepatitis B and TB reactivation.

## 1. Introduction

Inflammatory bowel disease (IBD) is a chronic inflammatory disease that affects the gastrointestinal tract. The 2 major types of IBD are Crohn’s disease (CD) and ulcerative colitis (UC). The exact cause of IBD remains unknown; however, genetic susceptibility [1], environmental factors, changes in intestinal microbiota [2,3], and dysregulated immune system [4] are all associated with the disease. In addition to intolerable symptoms, IBD is linked with several complications, such as perforation, strictures, abscess formation, fistulas, extraintestinal manifestations [5], and colorectal cancer [6,7].

Anti-inflammatory agents, immunosuppressants, and biologic agents are used to treat IBD [8,9]. Anti-inflammatory agents include corticosteroids and 5-aminosalicylates (5-ASAs), and immunosuppressants include thiopurine, cyclosporine, and methotrexate. Biologic agents include tumor necrosis factor (TNF)-α inhibitors and anti-integrin agents. The wide usage of anti-inflammatory agents and immunosuppressants and the availability of biological therapeutic agents have all contributed to the control of the course of IBD. Over the past decade, understanding of mechanisms underlying IBD has also led to vigorous development of biologic agents.

5-ASAs are preferred for the treatment of mild or moderately active proctitis as well as for first-line maintenance treatment of UC [10]. The benefits of 5-ASAs in the treatment of CD are limited [11], and they are not routinely used. Overall, 5-ASAs are safe, but they may cause GI-related symptoms (i.e., nausea, vomiting, loss of appetite, and abdominal pain). Corticosteroids are recommended in moderately to severely active localized ileocecal CD, extensive small bowel disease, refractory proctitis, and severely active UC [10,11]. However, corticosteroids may cause adverse effects, including osteoporosis, gastrointestinal bleeding, cataract, hypertension, and vulnerability to infection. Corticosteroids are used for induction therapy and are not effective for maintenance of remission in CD [11]. Steroid-free remission is also the goal of maintenance therapy for UC [10]. Thiopurine is recommended for patients with IBD who exhibit extensive disease or steroid dependency, and thiopurine should be considered for patients whose IBD has gone into remission after systemic steroid treatment [10,11]. However, thiopurine may induce dose-dependent adverse events such as myelosuppression and hepatotoxicity, and it may increase the risk of lymphoma [12,13]. Anti-TNF-α agents are superior to conventional therapies and are recommended for cases of active disease refractory to corticosteroids, moderate colitis refractory to thiopurine, and steroid dependency [10,11]. Anti-TNF-α agents are effective for both induction and maintenance therapy in CD and UC, and they also reduce IBD-related hospitalization and promote mucosal healing in UC induction [14,15]. Anti-TNF-α agents may cause injection site reactions and increase the risk of infection. In combination, thiopurine and anti-TNF-α agents are associated with increased risks of nonmelanoma skin cancers and lymphoma [13,16]. 

Considering the benefits and adverse effects of these medications, appropriate treatment durations and long-term outcomes remain controversial. For Asian populations, data are lacking in terms of long-term follow-up in large-scale studies of IBD-related medication usage and related safety concerns. In this retrospective study, we explored trends of medication usage from 2001 to 2015 and investigated medication-associated outcomes for patients with IBD by using data from a nationwide health insurance database. 

## 2. Materials and Methods

### 2.1. Data Collection

A nationwide cohort study of data from the Registry for Catastrophic Illness Patient Database (RCIPD), a subset of the Taiwan National Health Insurance Research Database (NHIRD), was conducted for the period from 1 January 2001, to 31 December 2015. In Taiwan, NHIRD-defined catastrophic illnesses exempt specific populations from health insurance fees. Because of their association with repetitive admissions and the need for chronic, careful care, CD and UC have been registered as catastrophic illnesses since 1997. When applying for the registration of a patient with one of these illnesses, health care providers are required to provide a clinical history record, endoscopic reports, imaging and histology results. After reviewing these records, the reviewers of the National Health Insurance (NHI) system approve or decline the application. Therefore, not all clinical IBD patients are registered. Regardless, for this study, the critical point is that all registered patients are confirmed patients. Comprehensive medical information, including each patient’s sex, age, disease diagnosis, history of surgery, and medication usage, was retrieved from the RCIPD. Before records of codes are released through the RCIPD, identification information is encrypted to maintain patient privacy. These data were sourced from the Health and Welfare Data Science Center, Ministry of Health and Welfare.

In the present study, we used International Classification of Diseases, Ninth Revision, Clinical Modification (ICD-9-CM) codes for the identification of UC (ICD-9-CM code: 556.9), CD (ICD-9-CM code: 555.9), pneumonia (ICD-9-CM codes: 482 and 486), urinary tract infection (ICD-9-CM code: 599.9), sepsis (ICD-9-CM code: 038), abdominal abscess (ICD-9-CM codes: 566 and 569.5), hepatitis B (ICD-9-CM codes: 070.2, 070.3, and V02.61), tuberculosis (TB; ICD-9-CM codes: 010–018), colorectal cancer (ICD-9-CM codes: 153 and 154), liver cancer (ICD-9-CM code: 155), lymphoma (ICD-9-CM codes: 200 and 202), skin cancer (ICD-9-CM codes: 172 and 173), hypertension (ICD-9-CM code: 401), diabetes (ICD-9-CM code: 250), and hyperlipidemia (ICD-9-CM code: 272). Pneumonia, urinary tract infection, sepsis, or abdominal abscess was confirmed if the relevant diagnosis was noted in patients’ discharge summaries. Hypertension, diabetes, or hyperlipidemia diagnosis was confirmed if the corresponding ICD-9-CM code was recorded more than 3 times in the outpatient medical record. Reactivation of hepatitis B was identified if a patient with hepatitis B history received lamivudine, entecavir, or tenofovir. For TB, reactivation was identified if patients with TB history received isoniazid, rifampicin, pyrazinamide, ethambutol, or other anti-TB agent. Operation codes were used for colectomy (73014B), exploratory laparotomy (75805B), and ileostomy (730158, 73016B, and 73017B). Hospitalizations due to accidents were excluded from the definition of hospitalization in this study.

The Taiwan Food and Drug Administration approved the use of the anti-TNF antibody for the treatment of CD in December 2007 and for the treatment of UC in February 2013. The NHI system has reimbursed patients for anti-TNF antibody treatment of CD since July 2011 and for that of UC since October 2016. In addition, the NHI has reimbursed patients for anti-integrin agents since November 2017, but we did not evaluate outcomes for anti-integrin agents in this study.

### 2.2. Statistical Analyses

Results are expressed as the mean, median, and range. The incidence rate is presented per 100,000 person-months. A Cox proportional hazards model was used to calculate hazard ratios (HRs) and 95% confidence intervals (CIs) from the RCIPD data. The infection and operation HRs associated with each medication were adjusted by sex, age, hypertension, diabetes, and hyperlipidemia. Analyses were performed using SAS (SAS, Cary, NC, USA), and a *p* value of <0.05 was considered significant.

## 3. Results

### 3.1. Patient Characteristics

A total of 3806 patients were registered for IBD in the RCIPD from 2001 to 2015. Among these patients, 919 (24.1%) were diagnosed with CD and 2887 (75.9%) were diagnosed with UC. The male population comprised 68.7% of the CD group and 61.9% of the UC group. The median age was 35 years in the CD group and 44 years in the UC group. IBD medications that had ever been prescribed to patients in the CD group were 5-ASAs (*n* = 754, 82.1%), thiopurine (*n* = 424, 46.1%), anti-TNF-α agents (*n* = 249, 27.1%), and corticosteroids (*n* = 726, 79%). In the UC group, IBD medications that had ever been used were 5-ASAs (*n* = 2636, 91.3%), thiopurine (*n* = 462, 16%), anti-TNF-α agents (*n* = 16, 0.6%), and corticosteroids (*n* = 2105, 72.9%; Table 1). A higher number of patients with CD used thiopurine than did that of patients with UC. The number of patients who used an anti-TNF-α agent for UC was relatively low because the NHI only began reimbursing patients for this medication in October 2016. 

### 3.2. Medication Trends among Patients with IBD

**Figure 1:**
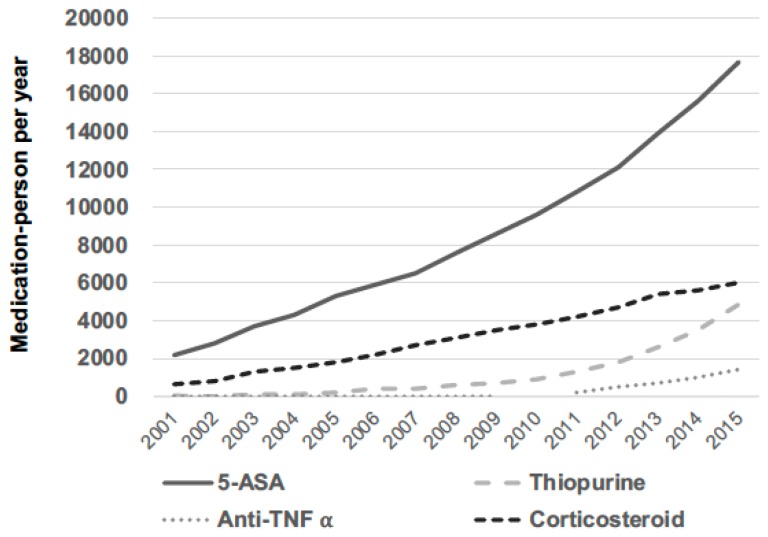
Annual prescribed IBD medication-person dosage.

In general, the most frequently used medication among Taiwanese patients with IBD was 5-ASAs, followed by corticosteroids. The least-used medication type was anti-TNF-α agents. The use of all 4 of these medications increased during the study period, and the rate of corticosteroid usage appeared to increase slowly over time (Figure 1).

### 3.3. Risks of Hospitalization, Operation, and Infection Associated with 5-ASA Treatment

We divided patients who had received 5-ASA treatment into 4 groups. The recommended daily dosage of 5-ASA was 2 g. Group 1 comprised patients who had received cumulative 5-ASA dosages of less than 0.1 g; this group served as the control. Group 2 was defined as the low accumulated dosage group (treatment with 2 g/day for less than 1.5 years) and comprised patients who had received cumulative 5-ASA dosages between 0.101 and 1095 g. Group 3 was defined as the medium accumulated dosage group (treatment with 2 g/day for 1.5–3 years) and comprised patients who had received cumulative 5-ASA dosages between 1095 and 2189 g. Finally, Group 4 comprised patients who had received 5-ASA treatment of 2 g/day for more than 3 years; this group was defined as the high accumulated dosage group. The patients with IBD who had received 5-ASA treatment exhibited relatively low risks of infection-related hospitalization or operation. High accumulated dosages of 5-ASA were correlated with decreased risks of infection-related hospitalization (HR = 0.6, 95% CI: 0.5–0.8), including that for pneumonia (HR = 0.5, 95% CI: 0.3–0.7), urinary tract infection (HR = 0.6, 95% CI: 0.4–0.8), and sepsis (HR = 0.6, 95% CI: 0.4–0.8); however, high accumulated dosage was also correlated with an increased risk of abdominal abscess (HR = 2.4, 95% CI: 1.1–5.3). In further subgroup analysis, we discovered that a decrease in infection-related hospitalization was evident for UC patients but not for CD patients; specifically, decreased risks of pneumonia (HR = 0.5, 95% CI: 0.3–0.8), urinary tract infection (HR = 0.6, 95% CI: 0.4–0.9), and sepsis (HR = 0.6, 95% CI: 0.4–0.9) were noted for the UC group. By contrast, among patients with CD, high accumulated dosage of 5-ASA was associated with higher risk of abdominal abscess (HR = 6.4, 95% CI: 1.9–20.9; Table 2). Patients in the high accumulated 5-ASA dosage group also exhibited relatively low risk of operation (HR = 0.5, 95% CI: 0.3–0.7), especially for colostomy (HR = 0.4, 95% CI: 0.3–0.7). In the subgroup analysis, the aforementioned results were similar for the UC group (HR = 0.4, 95% CI: 0.2–0.6) but not for the CD group (HR = 1, 95% CI: 0.6–1.8). No associations between 5-ASA treatment and risks of hepatitis B or TB infection were observed (Table 3). 

### 3.4. Risks of Hospitalization, Operation, and Infection Associated with Thiopurine Treatment

We divided patients who had received thiopurine treatment into 3 groups. The recommended minimum daily dosage of thiopurine was 50 mg. Group 1 comprised patients who had received cumulative thiopurine dosages of less than 100 mg; this group was defined as the control group. Patients assigned to Group 2 were those who had received cumulative thiopurine dosages between 101 and 18,250 mg, and this group was defined as the low accumulated dosage group (treatment with 50 mg/day for less than 1 year). Group 3 was defined as the high accumulated dosage group and comprised patients who had received thiopurine dosages of 50 mg/day for more than 1 year. Patients with IBD who had received either low or high accumulated dosages of thiopurine exhibited increased risks of infection-related hospitalization (HR = 2.3, 1.9–2.9 in the low accumulated dosage group; HR = 2.1, 1.7–2.7 in the high accumulated dosage group) including that for urinary tract infection, sepsis, and abdominal abscess. In the subgroup analysis, this finding was observed in the UC group but not in the CD group. Thiopurine treatment did not correlate with operation risk (HR = 1.0, 95% CI: 0.7–1.4). Treatment with a high accumulated dosage of thiopurine for patients with IBD was associated with increased risk of TB reactivation (HR = 3.6, 95% CI: 1.7–7.3). Additionally, in the subgroup analysis, treatment with a high accumulated dosage of thiopurine was associated with increased TB (HR = 4.1, 95% CI: 1.1–14.7) reactivation among patients with UC. Among patients in the CD group, even treatment with a low accumulated dosage of thiopurine was associated with increased hepatitis B (HR = 9.7, 95% CI: 2.1–44.1) reactivation (Table 2). 

### 3.5. Risks of Hospitalization, Operation, and Infection Associated with Anti-TNF-α Treatment

Patients who had received anti-TNF-α agents were divided into 3 groups. The recommended 1-year dosage of anti-TNF-α was 1280 mg. Group 1, the control group, comprised patients who had received cumulative anti-TNF-α dosages of less than 100 mg. Group 2 was defined as the low accumulated dosage group (treatment less than 1 year) and comprised patients who had received cumulative anti-TNF-α dosages between 101 and 1280 mg. Group 3, defined as the high accumulated dosage group, comprised patients who had received anti-TNF-α treatment for more than 1 year. Both the low and high accumulated dosage treatments with anti-TNF-α agents were associated with an increased risk of infection-related hospitalization (HR = 4.0, 95% CI: 2.9–5.5 in the low accumulated dosage group, HR = 3.3, 95% CI: 2.4–4.5 in the high accumulated dosage group). In patients with IBD who had received low accumulated dosages of anti-TNF-α agents, risks for pneumonia (HR = 2.2, 95% CI: 1.1–4.5), urinary tract infection (HR = 2.4, 95% CI: 1.3–4.5), sepsis (HR = 4.0, 95% CI: 2.6–6.1), and abdominal abscess (HR = 6.0, 95% CI: 3.4–10.5) increased. The incidence of colostomy rose among patients with IBD who had received high accumulated dosage of anti-TNF-α treatments (HR = 3.9, 95% CI: 2.4–6.4) in both the CD (HR= 2.1, 95% CI: 1.2–3.7) and UC groups (HR = 12.4, 95% CI: 3.0–51.3). The risks of hepatitis B (HR = 4.3, 95% CI: 1.5–12.7) and TB (HR = 5.1, 95% CI: 2.0–13.6) reactivation also increased for patients with IBD who were treated with anti-TNF-α, especially among those in the high accumulated dosage group. As few patients with UC were treated with anti-TNF-α agents in this study, none of them experienced hepatitis B or tuberculosis reactivation (Table 4).

### 3.6. Risks of Hospitalization, Operation, and Infection Associated with Corticosteroid Treatment

**Figure 2:**
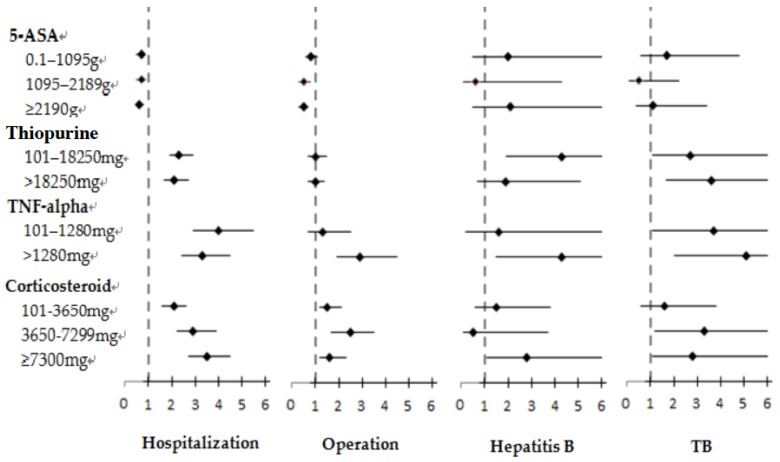
HRs and 95% CIs for hospitalization, operation, hepatitis B, and TB activation among patients with IBD in association with various medications. Dosage less than 100 mg is the reference group for each medication.

Corticosteroid-treated patients were also divided into 4 groups. The commonly used daily corticosteroid dosage was 10 mg. The corresponding group (Group 1) was set as the control group and comprised patients who had received cumulative corticosteroid dosages of less than 100 mg. Patients in Group 2 had received cumulative corticosteroid dosages between 101 and 3650 mg, and this group was defined as the low accumulated dosage group (treatment with 10 mg/day for less than 1 year). Group 3 was defined as the medium accumulated dosage group (treatment with 10 mg/day for 1–2 years) and comprised patients who had received cumulative corticosteroid dosages between 3650 and 7300 mg. Finally, Group 4 was the high accumulated dosage group and comprised patients who had received corticosteroid treatment of 10 mg/day for more than 2 years. Corticosteroid-treated IBD patients in all 3 dosage groups exhibited dosage-dependent increased risk of hospitalization (HR = 2.1, 95% CI: 1.6–2.6 in the low accumulated dosage group; HR = 2.9, 95% CI: 2.2–3.9 in the medium accumulated dosage group; HR = 3.5, 95% CI: 2.7–4.5 in the high accumulated dosage group). Risks of pneumonia (HR = 4.2, 95% CI: 2.7–6.6), urinary tract infection (HR = 2.8, 95% CI: 1.9–4.3), sepsis (HR = 3.7, 95% CI: 2.7–5.3), and abdominal abscess (HR = 3.4, 95% CI: 2.0–5.9) increased for patients in the high accumulated corticosteroid-dosage group. Increased risks of colostomy and ileostomy were also noted among these patients across low, medium, and high accumulated dosage groups. The results of the subgroup analysis revealed higher risks of colostomy (HR = 2.6, 1.4–4.8 in the medium-dosage group; HR = 1.8, 1.0–3.2 in the high-dosage group) and ileostomy (HR = 5.7, 2.7–12.3 in the medium-dosage group, HR = 2.7, 1.2–5.9 in the high-dosage group) among patients with UC but not among those with CD. The risks of hepatitis B (HR = 2.8, 95% CI: 1.1–7.2) and TB (HR = 2.8, 95% CI: 1.1–7.1) reactivation were both greater in correlation with higher corticosteroid dosages (Table 5). The HRs of hospitalization, operation, and infection for each medication are summarized in Figure 2 and Supplementary Figures S1 and S2. 

### 3.7. Risks of Tumor Incidence Associated with Various IBD Medications

Only a few patients in this study had diagnoses of a malignant tumor. We divided these patients into 2 groups: patients who had received cumulative medication dosages of less than 100 mg and those who had received more than 101 mg. Four tumor types (colorectal cancer, liver cancer, lymphoma, and skin cancer) were considered in this study. The results indicated no associations between treatment with 5-ASA, thiopurine, or anti-TNF-α agents and tumor development. However, patients who used corticosteroids exhibited an increased incidence risk of the 4 tumor types (HR = 3.85, 95% CI: 1.37–10.80). Subgroup analysis did not reveal any significant differences among results according to tumor type (Supplementary Tables S1−S4).

## 4. Discussion

With the increasing incidence and prevalence of IBD in Asia [17,18,19,20,21], IBD drug-related safety concerns require further attention. However, no large-scale studies with long-term follow-up for Asian populations have been conducted. Our study results indicated that 5-ASA may reduce risks of infection-related hospitalization and operation. Thiopurine, anti-TNF-α antibody, and steroids were all associated with increased risks of TB and hepatitis B reactivation, which are critical safety concerns in Asia. Additionally, in contrast to reports from Western countries, we did not observe associations between an increased risk of malignancy and thiopurine or anti-TNF-α antibody usage among patients with IBD [16,22], but steroid usage appeared to be associated with the risk of tumor development. 

5-ASA-treated patients with IBD, especially those with UC, had lower risks of infection-related hospitalization and operation. 5-ASA is effective in induction therapy and remission maintenance for mild-to-moderate UC, but its potential for CD treatment is limited [11,23]. In Taiwan, a high proportion of CD patients (82.1%) received 5-ASA treatment during the present study period. Treating CD patients with 5-ASA is not recommended in most of the guidelines. In this study, 5-ASA administration may have been relatively high because two-thirds of the CD cases in Taiwan involved colonic or ileocolonic locations and use of 5-ASA was associated with few side effects. Further analysis revealed that only 8.5% of CD patients received single 5-ASA treatment; the remaining patients received combination therapy. Previously, doctors in Taiwan administered immunomodulators relatively rarely. Furthermore, biologicals became available in Taiwan later than in Western countries. All these factors may have contributed to the relatively high proportion of CD patients treated with 5-ASA. 5-ASA exhibits an anti-inflammatory effect, the mechanism of which remains poorly understood. One hypothesis is that N-acetyl-5-ASA binds to peroxisome proliferator-activated receptor gamma, a nuclear receptor that modulates several key genes through mechanisms such as the nuclear factor-κB pathway [24,25,26]. A review of the literature did not reveal any reports indicating that 5-ASA can prevent infection. Patients who received 5-ASA usually presented with mild or well-controlled diseases, which would explain the relatively low risk of infection among these patients. In this study, 5-ASA was not associated with hepatitis B or TB reactivation. We observed a higher proportion of 5-ASA use among CD patients in our study cohort, which may have been attributable to the unavailability of budesonide in Taiwan. Most of the CD patients thus likely received initial treatment with 5-ASA because of its safety profile. The association of 5-ASA with an increased risk of abdominal abscess in CD patients may have reflected that the diseases were under treatment, resulting in more perforated CD complications. Therefore, our results support the usage of 5-ASA to improve outcomes in IBD treatment for patients with UC but not CD. 

In this study, the HR of infection was relatively high among patients with IBD who were on thiopurine. Thiopurine involves purine analogues that exhibit cytotoxic effects on dividing cells. Thiopurine suppresses immune response by inhibiting the clonal proliferation process of the adaptive immune response and deactivating T lymphocytes that cause inflammation [27,28]. In this study, thiopurine use was also associated with increased risks of TB and hepatitis B reactivation. Previous studies have revealed that exposure to thiopurine induces hepatitis B [29,30] and TB [31] reactivation. Current guidelines for management of hepatitis B and TB in patients with IBD do not emphasize the risks of thiopurine usage [11]; however, the increased risks of TB and hepatitis B associated with combined treatment with immunosuppressive agents (including thiopurine, steroids, and anti-TNF-α agents) has been reported [32]. Based on our study results, we suggest screening for hepatitis B and TB before prescribing steroids, immunosuppressants, or biologic agents to patients with IBD. Usage of thiopurine for more than 12 months has been reported to reduce the risk of colectomy among patients with elderly onset UC but not among those with onset of CD at an advanced age (>60 years at diagnosis) [33]. However, no relationship between thiopurine and operation risk was noted in our observation.

Anti-TNF-α agents predisposed patients with IBD to infection. Higher doses of the TNF-α inhibitor were also associated with increased risks of hepatitis B and TB reactivation among patients in this study. TNF-α is a type of proinflammatory cytokine. Correlations between anti-TNF-α agents and increased risks of serious infection (non-gastrointestinal bacterial infection requiring hospitalization) [34], and TB [35,36,37] and hepatitis B [38] reactivation have been reported. In the present study, anti-TNF-α agents were associated with an increased risk of colostomy because the disease activity of most patients exposed to the TNF-α inhibitor was severe.

We also observed that corticosteroid treatment was associated with an increased risk of non-gastrointestinal bacterial infections requiring hospitalization, including pneumonia, urinary tract infection, sepsis, and abdominal abscess. Additionally, higher steroid usage was correlated with increased risks of hepatitis B and TB reactivation. Glucocorticoids (GCs) are used to treat numerous diseases, and they cause various adverse effects. Cortisone and hydrocortisone are generally preferred for adrenal insufficiency replacement therapy. Corticosteroids are preferred for the treatment of many autoimmune or inflammatory diseases, whereas long-term GC usage increases infection risk. GCs suppress the immune system by affecting leukocyte migration, limiting chemotaxis of macrophages, and reducing the release of cytokines [39,40]. These mechanisms are consistent with our findings. In this study, corticosteroid treatment was also associated with increased risk of operation, because most patients treated with corticosteroids had severe colitis. 

Therapies for IBD have evolved in the past 2 decades, but the findings of the present study revealed that most of the patients with IBD in Taiwan received 5-ASA. For patients with UC, 5-ASA helped control disease activity and was associated with lower incidences of infection-related hospitalization and surgery. However, clinicians should be advised to change their treatment strategy for patients with CD; 5-ASA only exhibits a marginal effect on CD, and our results indicated that incidence of intraabdominal abscess increased when disease activity was not fully under control. A relatively low number of cases with therapy escalation were identified among the UC patients because of concerns regarding the adverse effects of immunosuppressants and because the NHI began reimbursing patients for anti-TNF-α agents in September 2016 [41]. The “treat to target” idea appeared to have been implemented during the present study period; we identified relatively high numbers of patients who had received immunomodulators and biologics. However, our finding of a high proportion of patients using steroids suggested that the “steroid sparing” concept may require further promotion. 

The Western Pacific region remains an endemic area for hepatitis B and TB infection. In this area, HBsAg prevalence was 5.26% [42], and 10.4 million people had TB in 2016. The Western Pacific region carries 17% of the global TB burden [43]. Screening for hepatitis B and TB should be performed prior to a patient’s usage of corticosteroids, immunomodulators, or biologic agents.

To our knowledge, this is the first long-term, large-scale study of an Asian population assessing outcomes associated with IBD medications. The strength of this study is that it involves analysis of nationwide population-based data with stringent criteria for IBD diagnosis (validated by catastrophic illness registration) and a long follow-up period (15 years). However, this study also has some limitations. First, this was a retrospective observational study. Second, certain types of information were not available in the database, including IBD activity, involved locations, and relevant laboratory data. Third, we assessed accumulated dosages instead of accurate doses for the analysis period. Fourth, we did not compare the effects of single and combined medication usages because categorization based on the various combinations would have resulted in groups comprising too few patients for analysis. A prospective randomized trial is required to confirm our results.

## 5. Conclusions

This retrospective nationwide database study indicated that 5-ASA usage was associated with decreased risks of hospitalization and operation for patients with IBD. The associated risk of operation increased among patients with IBD who were treated with corticosteroid or high accumulated dose of anti-TNF-α agents because the disease activity of these patients was usually severe. Treatments with thiopurine, corticosteroids, and anti-TNF-α agents were correlated with increases in the associated risks of infection-related hospitalization as well as hepatitis B and TB reactivation. The IBD medications analyzed in this study exhibited neither chemo-preventive nor promotional effects on tumor development. 

## Figures and Tables

**Table 1:** Basic characteristics and medication distribution of patients with IBD diagnosed from 2001 to 2015.

Variables	Crohn’s Disease, *n* = 919	Ulcerative Colitis, *n* = 2887
**Age of diagnosis, year**		
Mean (sd)	38.1 (18.5)	45.0 (15.6)
median (Q1–Q3)	35 (24–51)	44 (34–55)
**Age of diagnosis, *n* (%)**		
<20	128 (13.9)	115 (4.0)
20–39	415 (45.2)	1003 (34.7)
40–59	241 (26.2)	1251 (43.3)
>60	135 (14.7)	518 (17.9)
**Male, *n* (%)**	631 (68.7)	1787 (61.9)
Follow-up time, years		
mean(sd)	5.1 (4.2)	6.7 (4.2)
median (Q1–Q3)	3.9 (1.5–8.2)	6.5 (3.0–10.2)
5-aminosalicylic acid, *n* (%)	754 (82.1)	2636 (91.3)
Only	78	659
Others	676	1977
Thiopurine, *n* (%)	424 (46.1)	462 (16.0)
Only	8	0
Others	416	462
Anti-TNF a, *n* (%)	249 (27.1)	16 (0.6)
Only	0	16
Others	249	
Corticosteroid, *n* (%)	726 (79.0)	2105 (72.9)
Only	0	0
Others	726	2105

Cases ever used the drug were included in the calculation, regardless of the dose. Only refers to cases of single use of the drug; others refer to the drug used in combination with any of the other 3 drugs.

**Table 2:** Risks of operation, hospitalization, and related diseases associated with 5-aminosalicylic acid therapy among patients with IBD in Taiwan, 2001–2015.

Outcome		5–Aminosalicylic Acid (g)																									
		IBD							CD									UC									
		<0.1	0.1–1095		1095–2189		≥2190		<0.1	0.1–1095			1095–2189		≥2190			<0.1	0.1–1095			1095–2189			≥2190		
Follow person		417	1715		636		1038		166	402			143		208			251	1313			493			830		
**4 causes of hospitalization** ^‡^																											
**IR**		413.4	256.2		235.4		208.3		514.7	626.8			501.7		475.2			368.5	193.0			176.0			154.4		
**HR (95% CI)**		1.0	0.7 *	(0.5–0.8)	0.7 *	(0.5–0.9)	0.6 *	(0.5–0.8)	1.0	1.1	(0.8–1.6)		1.1	(0.7–1.6)	1.1	(0.8–1.7)		1.0	0.6 *	(0.5–0.9)		0.6 *	(0.4–0.9)		0.6 *	(0.4–0.8)	
Pneumonia	IR	154.6	88.7		86.3		50.1		196.6	141.0			108.9		93.0			135.6	78.9			80.7			40.1		
	HR	1.0	0.7	(0.5–1.0)	0.8	(0.5–1.2)	0.5 *	(0.3–0.7)	1.0	0.7	(0.4–1.4)		0.7	(0.3–1.5)	0.6	(0.3–1.3)		1.0	0.8	(0.5–1.3)		0.9	(0.6–1.6)		0.5 *	(0.3–0.8)	
Urinary tract infection	IR	160.9	86.8		83.8		67.0		178.5	160.0			141.2		112.2			152.6	73.2			69.7			56.6		
	HR	1.0	0.7 *	(0.5–0.9)	0.7	(0.5–1.2)	0.6 *	(0.4–0.8)	1.0	0.9	(0.5–1.6)		0.9	(0.4–1.9)	0.8	(0.4–1.6)		1.0	0.7	(0.4–1.1)		0.8	(0.4–1.3)		0.6 *	(0.4–0.9)	
Sepsis	IR	199.3	144.3		106.5		94.6		253.0	370.3			233.6		161.2			174.6	104.6			75.6			79.2		
	HR	1.0	0.8	(0.6–1.1)	0.6 *	(0.4–0.9)	0.6 *	(0.4–0.8)	1.0	1.3	(0.8–2.1)		1.0	(0.5–1.8)	0.8	(0.4–1.4)		1.0	0.8	(0.5–1.2)		0.6	(0.4–1.0)		0.6 *	(0.4–0.9)	
Abdominal abscess	IR	23.9	37.7		42.7		54.2		31.4	105.1			132.2		177.9			20.3	25.1			21.2			26.9		
	HR	1.0	1.5	(0.7–3.4)	1.7	(0.7–3.9)	2.4 *	(1.1–5.3)	1.0	2.9	(0.9–10.0)		3.9 *	(1.1–13.9)	6.4 *	(1.9–20.9)		1.0	1.1	(0.4–3.2)		0.9	(0.3–2.9)		1.2	(0.4–3.5)	
**4 types of operation** ^§^																											
**IR**		189.2	159.6		88.0		65.8		222.2	366.3			136.7		146.7			172.6	121.9			76.4			48.1		
**HR (95% CI)**		1.0	0.8	(0.6–1.1)	0.5 *	(0.3–0.8)	0.5 *	(0.3–0.7)	1.0	1.3	(0.8–2.2)		0.7	(0.3–1.5)	1.0	(0.6–1.8)		1.0	0.7	(0.5–1.0)		0.5 *	(0.3–0.8)		0.4 *	(0.2–0.6)	
Colectomy	IR	10.1	5.4		8.4		1.9		10.2	17.3			20.9		0.0			10.0	3.2			5.3			2.4		
	HR	1.0	0.6	(0.1–2.4)	1.1	(0.2–5.2)	0.3	(0.1–1.9)	1.0	1.4	(0.1–13.9)		3.6	(0.3–43.2)	–			1.0	0.4	(0.1–2.4)		0.8	(0.1–5.8)		0.4	(0.1–2.7)	
Colostomy	IR	105.9	105.7		45.2		33.5		127.1	260.7			53.5		73.3			95.3	77.3			43.1			24.3		
	HR	1.0	1.0	(0.6–1.4)	0.5 *	(0.3–0.8)	0.4 *	(0.3–0.7)	1.0	1.6	(0.9–3.1)		0.5	(0.2–1.4)	0.9	(0.4–1.9)		1.0	0.8	(0.5–1.4)		0.5 *	(0.2–0.9)		0.3 *	(0.2–0.6)	
Exploratory laparotomy	IR	27.1	16.4		4.2		9.7		74.0	58.2			10.4		36.2			5.0	8.6			2.6			3.6		
	HR	1.0	0.6	(0.2–1.3)	0.2 *	(0.0–0.7)	0.4	(0.2–1.1)	1.0	0.6	(0.2–1.7)		0.2	(0.0–1.3)	0.7	(0.3–2.1)		1.0	1.6	(0.2–13.1)		0.5	(0.0–8.0)		0.8	(0.1–7.5)	
Ileostomy	IR	52.0	43.8		40.8		23.6		41.9	63.2			77.2		25.7			57.0	40.0			32.0			23.2		
	HR	1.0	0.8	(0.5–1.5)	0.8	(0.4–1.7)	0.6	(0.3–1.1)	1.0	1.2	(0.4–3.8)		1.9	(0.6–6.7)	1.0	(0.3–3.6)		1.0	0.7	(0.3–1.4)		0.6	(0.3–1.3)		0.5	(0.2–1.0)	
**2 types of disease** ^¶^																											
Hepatitis B	IR	6.7	14.6		4.2		13.6		0.0	40.3			10.5		5.0			10.0	9.7			2.6			15.6		
	HR	1.0	2.0	(0.5–8.8)	0.6	(0.1–4.3)	2.1	(0.5–9.5)	–	–		.	–		–			1.0	0.9	(0.2–4.3)		0.2	(0.0–2.8)		1.6	(0.4–7.0)	
TB	IR	13.5	21.9		6.3		12.6		31.3	76.4			20.9		35.5			5.0	11.9			2.6			7.2		
	HR	1.0	1.7	(0.6–4.8)	0.5	(0.1–2.2)	1.1	(0.4–3.4)	1.0	1.9	(0.5–6.8)		0.6	(0.1–3.8)	1.3	(0.3–5.1)		1.0	3.3	(0.4–25.6)		0.7	(0.0–11.5)		2.1	(0.2–17.5)	

Entire model adjusted by gender, age, hypertension (ICD–9–CM code: 401), diabetes (ICD–9–CM code: 250) and hyperlipidemia (ICD–9–CM code: 272) (disease confirmed based on >3 visits to the outpatient department). IR = 1/105 person–month. ‡ Including pneumonia (ICD-9-CM codes: 482 and 486), urinary tract infection (ICD-9-CM code: 599.0), sepsis (ICD-9-CM code: 038), abdominal wall abscess (ICD–9–CM codes: 566 and 569.5) (confirmed by diagnosis at discharge). § Including colectomy (73014B), colostomy (73022B), exploratory laparotomy (75805B), ileostomy (73015B, 73016B, and 73017B). ¶ Hepatitis B (ICD-9-CM codes: 070.20, 070.21, 070.22, 070.23, 070.30, 070.31, 070.32, 070.33. and v02.61) and drug (lamivudine, tenofovir, and entecavir). TB (ICD-9-CM code: 010–018) and drug (isoniazid, ethambutol, rifampin, and pyrazinamide). * *p* < 0.05.

**Table 3:** Risks of operation, hospitalization, and related diseases associated with thiopurine therapy among patients with IBD in Taiwan, 2001–2015.

Outcome	Thiopurine (mg)																							
		IBD								CD								UC						
		<100	101–18,250				>18,250			<100		101–18,250			1,095,000–2,189,999			<100	101–18,250			>18,250		
Follow person		2928	437			441				498	177				244			2430	260			197		
**4 causes of hospitalization** ^‡^																								
**IR**		210.9	496.6			378.1				518.4	796.0				457.9			166.4	384.4			302.8		
**HR** **(95% CI)**		1.0	2.3 *	(1.9–2.9)		2.1 *		(1.7–2.7)		1.0	1.2		(0.9–1.7)		1.0	(0.7–1.3)		1.0	2.6 *	(2.0–3.5)		2.3 *	(1.7–3.2)	
Pneumonia	IR	80.5	95.2			73.5				152.6	113.3				82.9			68.9	87.8			64.1		
	HR	1.0	1.5	(1.0–2.4)		1.4		(0.9–2.2)		1.0	0.8		(0.4–1.6)		0.7	(0.4–1.3)		1.0	1.8 *	(1.0–3.0)		1.3	(0.7–2.6)	
Urinary tract infection	IR	78.2	167.4			87.9				155.9	208.3				89.7			65.8	150.8			86.1		
	HR	1.0	2.5 *	(1.8–3.6)		1.9 *		(1.2–2.8)		1.0	1.1		(0.6–2.1)		0.8	(0.4–1.4)		1.0	3.1 *	(2.0–4.7)		2.2 *	(1.2–3.9)	
Sepsis	IR	109.4	233.2			167.9				256.8	392.6				182.0			86.2	169.4			153.8		
	HR	1.0	2.2 *	(1.6–3.0)		1.9 *		(1.4–2.6)		1.0	1.3		(0.8–2.0)		0.8	(0.5–1.3)		1.0	2.3 *	(1.6–3.5)		2.3 *	(1.5–3.5)	
Abdominal abscess	IR	28.2	116.4			98.3				84.3	243.2				145.4			19.1	66.2			52.0		
	HR	1.0	3.2 *	(2.0–5.0)		2.6 *		(1.7–4.0)		1.0	2.2 *		(1.2–4.2)		1.5	(0.8–2.6)		1.0	3.0 *	(1.5–6.0)		2.3 *	(1.1–5.0)	
**4 types of operation** ^§^																								
**IR**		114.1	144.5			113.4				247.2	298.0				149.2			93.0	85.1			78.8		
**HR** **(95% CI)**		1.0	1.0	(0.7–1.5)		1.0		(0.7–1.4)		1.0	0.8		(0.5–1.3)		0.7	(0.4–1.1)		1.0	0.8	(0.5–1.5)		0.9	(0.5–1.6)	
Colectomy	IR	5.6	4.0			3.1				15.0	0.0				6.2			4.0	5.7			0.0		
	HR	1.0	0.6	(0.1–4.3)		0.7		(0.1–5.3)		1.0	–				0.6	(0.1–5.2)		1.0	1.2	(0.1–9.3)		–		
Colostomy	IR	68.3	88.9			64.1				145.8	206.4				83.1			55.7	41.6			45.0		
	HR	1.0	1.1	(0.7–1.7)		1.0		(0.6–1.6)		1.0	1.0		(0.5–1.8)		0.6	(0.3–1.2)		1.0	0.7	(0.3–1.5)		0.9	(0.4–1.9)	
Exploratory laparotomy	IR	12.4	16.3			15.8				51.8	42.7				31.8			6.0	5.7			0.0		
	HR	1.0	0.9	(0.3–2.6)		1.0		(0.4–2.7)		1.0	0.5		(0.1–1.7)		0.6	(0.2–1.6)		1.0	0.8	(0.1–6.6)		–		
Ileostomy	IR	38.0	37.2			28.6				61.6	56.7				18.8			34.1	29.2			38.6		
	HR	1.0	0.8	(0.4–1.6)		0.8		(0.4–1.5)		1.0	0.6		(0.2–1.7)		0.3	(0.1–1.1)		1.0	0.8	(0.3–2.0)		1.2	(0.5–2.7)	
**2 types of disease** ^¶^																								
Hepatitis B	IR	8.5	36.9			15.6				9.0	70.4				6.2			8.5	23.1			25.3		
	HR	1.0	4.3 *	(1.9–9.7)		1.9		(0.7–5.1)		1.0	9.7 *		(2.1–44.1)		0.8	(0.1–8.0)		1.0	2.5	(0.8–7.6)		2.8	(0.9–8.4)	
TB	IR	11.1	28.4			34.7				42.7	41.6				50.3			6.0	23.0			19.0		
	HR	1.0	2.7 *	(1.1–6.2)		3.6 *		(1.7–7.3)		1.0	0.8		(0.2–2.7)		1.3	(0.5–3.1)		1.0	4.8 *	(1.5–15.2)		4.1 *	(1.1–14.7)	

‡ Including pneumonia (ICD-9-CM codes: 482 and 486), urinary tract infection (ICD-9-CM code: 599.0), sepsis (ICD-9-CM code: 038), abdominal wall abscess (ICD–9–CM codes: 566 and 569.5) (confirmed by diagnosis at discharge). § Including colectomy (73014B), colostomy (73022B), exploratory laparotomy (75805B), ileostomy (73015B, 73016B, and 73017B). ¶ Hepatitis B (ICD-9-CM codes: 070.20, 070.21, 070.22, 070.23, 070.30, 070.31, 070.32, 070.33. and v02.61) and drug (lamivudine, tenofovir, and entecavir). TB (ICD-9-CM code: 010–018) and drug (isoniazid, ethambutol, rifampin, and pyrazinamide). * *p* < 0.05.

**Table 4:** Risks of operation, hospitalization, and related diseases associated with anti-TNF-α therapy among patients with IBD in Taiwan, 2001–2015.

Outcome		Anti-TNF-α Agent (mg)																							
		IBD								CD								UC							
		<100	101–1280				>1280			<100		101–1280			>1280			<100		101–1280			1280		
Follow person		3545	134			127				673	125				121			2872	9				6		
**4 causes of hospitalization** ^‡^																									
**IR**		228.1	1024			565.7				479.8	970.0				566.4			187.6	1782				551.3		
**HR (95% CI)**		1.0	4.0 *	(2.9–	5.5)	3.3 *		(2.4–	4.5)	1.0	1.6 *		(1.2–	2.4)	1.4	(1.0–	2.0)	1.0	5.9 *		(2.4–	14.4)	4.9 *	(1.2–	19.8)
Pneumonia	IR	80.6	147.2			52.2				142.2	139.4				55.0			69.9	242.0				0.0		
	HR	1.0	2.2 *	(1.1–	4.5)	1.3		(0.5–	3.1)	1.0	1.0		(0.4–	2.2)	0.5	(0.2–	1.3)	1.0	2.2		(0.3–	16.3)			
Urinary tract infection	IR	83.6	182.4			119.9				150.2	159.8				102.1			71.8	419.6				551.3		
	HR	1.0	2.4 *	(1.3–	4.5)	3.2 *		(1.7–	6.0)	1.0	0.8		(0.4–	1.8)	1.1	(0.5–	2.3)	1.0	6.0 *		(1.5–	24.6)	28.6 *	(6.8–	119.4)
Sepsis	IR	117.0	486.3			188.7				239.7	468.9				199.4			95.8	657.4				0.0		
	HR	1.0	4.0 *	(2.6–	6.1)	2.3 *		(1.4–	3.9)	1.0	1.7 *		(1.0–	2.7)	1.1	(0.6–	1.8)	1.0	5.1 *		(1.6–	16.2)			
Abdominal abscess	IR	31.7	302.1			241.4				78.2	282.5				243.2			23.5	551.1				210.3		
	HR	1.0	6.0 *	(3.4–	10.5)	5.4 *		(3.3–	8.8)	1.0	2.9 *		(1.5–	5.6)	2.9 *	(1.7–	5.1)	1.0	15.1 *		(3.6–	63.8)	5.8	(0.8–	42.5)
**4 types of operation** ^§^																									
**IR**		108.6	210.4			308.7				210.0	233.1				295.8			90.9	0.0				619.7		
**HR (95% CI)**		1.0	1.3	(0.7–	2.5)	2.9 *		(1.9–	4.5)	1.0	0.8		(0.4–	1.6)	1.8 *	(1.1–	2.8)	1.0	0.0				6.5 *	(1.6–	26.5)
Colectomy	IR	5.1	17.9			0.0				11.8	19.7				0.0			3.9	0.0				0.0		
	HR	1.0	2.5	(0.3–	19.6)	–				1.0	1.6		(0.2–	14.1)	–			1.0	–				–		
Colostomy	IR	63.9	108.5			218.5				123.2	119.6				203.1			53.2	0.0				619.7		
	HR	1.0	1.3	(0.6–	2.9)	3.9 *		(2.4–	6.4)	1.0	0.8		(0.3–	1.8)	2.1 *	(1.2–	3.7)	1.0	–				12.4 *	(3.0–	51.3)
Exploratory laparotomy	IR	12.4	55.8			10.3				50.6	61.7				10.8			5.6	0.0				0.0		
	HR	1.0	2.3	(0.7–	7.6)	0.7		(0.1–	5.0)	1.0	0.8		(0.2–	2.7)	0.2	(0.0–	1.9)	1.0	–				–		
Ileostomy	IR	36.0	35.9			65.4				45.5	39.5				69.0			34.2	0.0				0.0		
	HR	1.0	0.7	(0.2–	2.7)	1.9		(0.8–	4.4)	1.0	0.6		(0.1–	2.6)	1.9	(0.8–	5.0)	1.0	–				–		
**2 types of disease** ^¶^																									
Hepatitis B	IR	10.5	17.8			41.5				9.4	19.6				43.7			10.7	0.0				0.0		
	HR	1.0	1.6	(0.2–	11.9)	4.3 *		(1.5–	12.7)	1.0	2.6		(0.3–	25.3)	8.3 *	(1.8–	38.9)	1.0	–				–		
TB	IR	13.1	54.0			52.7				40.6	59.5				55.6			8.2	0.0				0.0		
	HR	1.0	3.7 *	(1.1–	12.0)	5.1 *		(2.0–	13.6)	1.0	1.1		(0.3–	3.9)	1.6	(0.6–	4.3)	1.0	–				–		

‡ Including pneumonia (ICD-9-CM codes: 482 and 486), urinary tract infection (ICD-9-CM code: 599.0), sepsis (ICD-9-CM code: 038), abdominal wall abscess (ICD–9–CM codes: 566 and 569.5) (confirmed by diagnosis at discharge). § Including colectomy (73014B), colostomy (73022B), exploratory laparotomy (75805B), ileostomy (73015B, 73016B, and 73017B). ¶ Hepatitis B (ICD-9-CM codes: 070.20, 070.21, 070.22, 070.23, 070.30, 070.31, 070.32, 070.33. and v02.61) and drug (lamivudine, tenofovir, and entecavir). TB (ICD-9-CM code: 010–018) and drug (isoniazid, ethambutol, rifampin, and pyrazinamide). * *p* < 0.05.

**Table 5:** Risks of operation, hospitalization, and related diseases associated with corticosteroid therapy among patients with IBD in Taiwan, 2001–2015.

Outcome		Corticosteroid (mg)																														
		IBD										CD											UC									
		<100	101–3650			3650–7299			≥7300			<100	101–3650				3650–7299			≥7300			<100	101–3650			3650–7299			≥7300		
Follow person		1257	1571			357			621			246		389			97			187			1011	1182			260			434		
**4 causes of hospitalization** ^‡^																																
**IR**		120.3	261.8			355.8			383.6			383.1		577.2			800.7			511.3			82.3	201.1			257.5			334.7		
**HR (95% CI)**		1.0	2.1 *	(1.6–	2.6)	2.9 *	(2.2–	3.9)	3.5 *	(2.7–	4.5)	1.0		1.4 *	(1.0–	2.0)	2.2 *	(1.4–	3.4)	1.7 *	(1.1–	2.5)	1.0	2.3 *	(1.7–	3.1)	2.9 *	(2.0–	4.2)	4.2 *	(3.1–	5.7)
Pneumonia	IR	31.0	93.3			103.3			117.8			61.2		155.7			139.5			134.4			26.3	80.2			93.6			111.0		
	HR	1.0	2.7 *	(1.8–	4.2)	3.0 *	(1.8–	5.2)	4.2 *	(2.7–	6.6)	1.0		2.3 *	(1.0–	5.3)	2.7	(1.0–	7.4)	3.1 *	(1.3–	7.3)	1.0	2.7 *	(1.6–	4.4)	2.8 *	(1.5–	5.3)	4.1 *	(2.4–	7.0)
Urinary tract infection	IR	43.1	97.3			150.3			100.0			107.6		154.6			266.4			115.6			33.2	85.0			120.8			93.4		
	HR	1.0	2.1 *	(1.4–	3.0)	3.6 *	(2.3–	5.7)	2.8 *	(1.9–	4.3)	1.0		1.2	(0.6–	2.3)	2.6 *	(1.2–	5.6)	1.4	(0.7–	2.8)	1.0	2.3 *	(1.5–	3.6)	3.4 *	(1.9–	6.0)	3.1 *	(1.9–	5.2)
Sepsis	IR	56.5	130.9			184.3			190.8			180.3		288.7			321.6			237.1			37.4	98.5			149.3			171.9		
	HR	1.0	2.2 *	(1.6–	3.1)	3.3 *	(2.2–	4.9)	3.7 *	(2.7–	5.3)	1.0		1.5	(0.9–	2.5)	2.0 *	(1.1–	3.9)	1.8 *	(1.0–	3.1)	1.0	2.4 *	(1.6–	3.7)	3.7 *	(2.2–	6.2)	4.6 *	(2.9–	7.2)
Abdominal abscess	IR	22.7	33.4			67.9			79.4			99.1		101.8			253.0			118.4			11.0	18.8			23.4			63.3		
	HR	1.0	1.6	(0.9–	2.7)	2.8 *	(1.5–	5.4)	3.4 *	(2.0–	5.9)	1.0		1.1	(0.5–	2.2)	2.5 *	(1.1–	5.7)	1.4	(0.7–	3.0)	1.0	1.8	(0.8–	4.3)	2.1	(0.7–	6.3)	5.7 *	(2.6–	12.7)
**4 types of operation** ^§^																																
**IR**		85.3	125.4			203.6			107.1			269.4		234.5			249.8			176.0			57.1	102.3			190.9			80.3		
**HR (95% CI)**		1.0	1.5 *	(1.2–	2.1)	2.5 *	(1.7–	3.5)	1.6 *	(1.2–	2.3)	1.0		0.9	(0.6–	1.5)	1.1	(0.6–	2.1)	1.2	(0.7–	2.0)	1.0	1.9 *	(1.3–	2.8)	3.4 *	(2.1–	5.3)	1.7 *	(1.1–	2.8)
Colectomy	IR	10.7	3.4			3.6			1.6			35.0		4.7			0.0			5.5			6.9	3.1			4.6			0.0		
	HR	1.0	0.3	(0.1–	1.0)	0.4	(0.0–	3.0)	0.2	(0.0–	1.7)	1.0		0.2	(0.0–	1.4)	–			0.4	(0.0–	4.0)	1.0	0.4	(0.1–	1.9)	0.8	(0.1–	6.6)	–		
Colostomy	IR	53.3	75.9			105.1			64.6			167.6		138.6			167.3			99.6			35.1	62.3			88.6			50.3		
	HR	1.0	1.5 *	(1.1–	2.2)	2.1 *	(1.3–	3.4)	1.6 *	(1.0–	2.5)	1.0		0.9	(0.5–	1.6)	1.2	(0.5–	2.6)	1.1	(0.5–	2.1)	1.0	1.9 *	(1.2–	3.0)	2.6 *	(1.4–	4.8)	1.8 *	(1.0–	3.2)
Exploratory laparotomy	IR	10.7	11.9			25.7			13.3			34.9		47.6			70.8			40.1			6.9	4.1			13.9			2.3		
	HR	1.0	1.2	(0.5–	2.7)	2.4	(0.9–	6.5)	1.4	(0.6–	3.8)	1.0		1.4	(0.4–	4.6)	2.2	(0.5–	8.9)	1.9	(0.5–	6.5)	1.0	0.6	(0.2–	2.4)	1.9	(0.5–	8.1)	0.4	(0.0–	3.1)
Ileostomy	IR	18.0	46.8			65.7			31.8			35.3		87.3			0.0			28.2			15.2	38.0			84.8			33.3		
	HR	1.0	2.8 *	(1.6–	4.9)	3.9 *	(1.9–	7.8)	2.3 *	(1.2–	4.6)	1.0		2.7	(0.9–	8.0)	–			1.4	(0.4–	5.3)	1.0	2.7 *	(1.4–	5.3)	5.7 *	(2.7–	12.3)	2.7 *	(1.2–	5.9)
**2 types of disease** ^¶^																																
Hepatitis B	IR	8.3	11.8			3.7			19.8			17.3		18.8			17.4			11.0			6.9	10.3			0.0			23.5		
	HR	1.0	1.5	(0.6–	3.8)	0.5	(0.1–	3.7)	2.8 *	(1.1–	7.2)	1.0		1.5	(0.3–	8.4)	1.5	(0.1–	16.7)	1.1	(0.2–	8.4)	1.0	1.6	(0.5–	4.7)	–			3.6 *	(1.2–	10.5)
TB	IR	8.3	13.6			29.3			21.5			34.6		53.1			69.6			33.6			4.1	5.2			18.6			16.5		
	HR	1.0	1.6	(0.6–	3.8)	3.3 *	(1.2–	9.2)	2.8 *	(1.1–	7.1)	1.0		1.5	(0.5–	4.7)	2.5	(0.6–	10.2)	1.4	(0.4–	5.0)	1.0	1.1	(0.3–	4.6)	3.6	(0.8–	16.3)	3.7	(0.9–	14.4)

‡ Including pneumonia (ICD-9-CM codes: 482 and 486), urinary tract infection (ICD-9-CM code: 599.0), sepsis (ICD-9-CM code: 038), abdominal wall abscess (ICD–9–CM codes: 566 and 569.5) (confirmed by diagnosis at discharge). § Including colectomy (73014B), colostomy (73022B), exploratory laparotomy (75805B), ileostomy (73015B, 73016B, and 73017B). ¶ Hepatitis B (ICD-9-CM codes: 070.20, 070.21, 070.22, 070.23, 070.30, 070.31, 070.32, 070.33. and v02.61) and drug (lamivudine, tenofovir, and entecavir). TB (ICD-9-CM code: 010–018) and drug (isoniazid, ethambutol, rifampin, and pyrazinamide). * *p* < 0.05.

## Supplementary Materials

The supplementary materials are available online at http://www.mdpi.com/2077-0383/7/11/394/s1. Figure S1: HR and 95% CIs for hospitalization, operation, hepatitis B and TB activation among CD patients with different medication, Figure S2: HR and 95% CIs for hospitalization, operation, hepatitis B and TB activation among UC patients with different medication, Table S1: Risk of tumor with 5-aminosalicylic acid therapy among IBD patients in Taiwan, 2001–2015, Table S2: Risk of tumor with thiopurine therapy among IBD patients in Taiwan, 2001–2015, Table S3: Risk of tumor with anti-TNF therapy among IBD patients in Taiwan, 2001–2015, Table S4: Risk of tumor with Corticosteroid therapy among IBD patients in Taiwan, 2001–2015.
