# Supplementary material for: Trends of Medication Usage and Associated Outcomes for Taiwanese Patients with Inflammatory Bowel Disease from 2001 to 2015
Source: J Clin Med. 2018 Oct 27;7(11):394. doi: 10.3390/jcm7110394 (PMC6262469; doi:10.3390/jcm7110394)
Supplement: Supplementary file 1 [file jcm-07-00394-s001.pdf]

Supplementary Materials

Supplement Table 1. Risk of tumor with 5-aminosalicylic acid therapy among IBD patients in Taiwan, 2001-2015.

	5-aminosalicylic acid (mg)								
	IBD			CD			UC		
**Outcome**	**<100**	**>=101**		**<100**	**>=101**		**<100**	**>=101**	
Follow person	417	3389		166	753		251	2636	
**4 types of tumor [a]**									
IR (1/10^5 person-month)	16.78	14.57		10.22	12.75		19.99	14.97	
HR (95% CI)	1.00	1.08	(0.42- 2.75)	1.00	1.58	(0.18- 13.73)	1.00	0.98	(0.34- 2.82)
**CRC**									
IR	6.69	5.74		0.00	6.37		9.96	5.60	
HR (95% CI)	1.00	0.93	(0.21- 4.12)	-	-		1.00	0.58	(0.13- 2.69)
**Lymphoma**									
IR	3.35	4.59		10.22	4.24		0.00	4.66	
HR (95% CI)	1.00	1.68	(0.22- 13.04)	1.00	0.40	(0.04- 4.49)	-	-	
**Liver cancer**									
IR	6.71	4.21		0.00	2.12		9.99	4.66	
HR (95% CI)	1.00	0.89	(0.19- 4.09)	-	-		1.00	0.67	(0.14- 3.16)
**Skin cancer**									
IR	3.35	1.15		10.22	2.12		0.00	0.93	
HR (95% CI)	1.00	0.48	(0.05- 4.85)	1.00	0.27	(0.01- 10.56)	-	-	

All model adjusted by gender, age, hypertension (ICD-9-CM code: 401), diabetes (ICD-9-CM code: 250) and hyperlipidemia (ICD-9-CM code: 272) (disease confirmed by visiting out-patient department for more than 3 times). IR=1/10^5 person-month. * $p<0.05$.

a. Including Colon/Rectum cancer (ICD-9-CM code: 153, 154), Liver cancer (ICD-9-CM code: 155), Lymphoma (ICD-9-CM code: 200, 202), skin cancer (ICD-9-CM code: 172, 173) (Confirmed by Registry for Catastrophic Illness Patient Database (RCIPD)).

Supplement Table 2. Risk of tumor with thiopurine therapy among IBD patients in Taiwan, 2001-2015.

	Thiopurine (mg)								
	IBD			CD			UC		
**Outcome**	**<100**	**>=101**		**<100**	**>=101**		**<100**	**>=101**	
Follow person	2928	878		498	421		2430	457	
**4 types of tumor [a]**									
IR (1/10^5 person-month)	14.96	14.12		14.99	8.51		14.96	18.08	
HR (95% CI)	1.00	1.21	(0.55- 2.64)	1.00	0.69	(0.13- 3.66)	1.00	1.50	(0.62- 3.66)
**CRC**									
IR	5.97	5.29		8.98	0.00		5.47	9.03	
HR (95% CI)	1.00	1.20	(0.34- 4.28)	1.00	-		1.00	2.16	(0.59- 7.95)
**Lymphoma**									
IR	4.26	5.28		5.98	4.25		3.98	6.00	
HR (95% CI)	1.00	1.26	(0.34- 4.71)	1.00	0.63	(0.06- 7.17)	1.00	1.57	(0.33- 7.61)
**Liver cancer**									
IR	4.69	3.52		0.00	4.25		5.47	3.00	
HR (95% CI)	1.00	1.14	(0.25- 5.31)	-	-		1.00	0.73	(0.09- 5.80)
**Skin cancer**									
IR	1.28	1.76		2.99	4.25		0.99	0.00	
HR (95% CI)	1.00	1.68	(0.17- 16.93)	1.00	13.97	(0.10- 2009)	1.00	-	

The analysis settings were the same as Supplement Table 1

Supplement Table 3. Risk of tumor with anti-TNF α therapy among IBD patients in Taiwan, 2001-2015.

	anti-TNF α agent (mg)								
	IBD			CD			UC		
**Outcome**	**<100**	**>=101**		**<100**	**>=101**		**<100**	**>=101**	
Follow person	3545	261		673	246		2872	15	
**4 types of tumor** [a]									
IR (1/10^5 person-month)	15.26	6.52		14.12	6.97		15.47	0.00	
HR (95% CI)	1.00	0.56	(0.08- 4.13)	1.00	0.61	(0.07- 5.23)	1.00	-	
**CRC**									
IR	5.80	6.52		4.70	6.97		6.00	0.00	
HR (95% CI)	1.00	1.66	(0.21- 12.98)	1.00	1.39	(0.12- 16.03)	1.00	-	
**Lymphoma**									
IR	4.71	0.00		7.04	0.00		4.28	0.00	
HR (95% CI)	1.00	-		1.00	-		1.00	-	
**Liver cancer**									
IR	4.71	0.00		2.35	0.00		5.14	0.00	
HR (95% CI)	1.00	-		1.00	-		1.00	-	
**Skin cancer**									
IR	1.45	0.00		4.69	0.00		0.86	0.00	
HR (95% CI)	1.00	-		1.00	-		1.00	-	

The analysis settings were the same as Supplement Table 1

Supplement Table 4. Risk of tumor with Corticosteroid therapy among IBD patients in Taiwan, 2001-2015.

	**Corticosteroid therapy (mg)**								
	**IBD**			**CD**			**UC**		
**Outcome**	**<100**	**>=101**		**<100**	**>=101**		**<100**	**>=101**	
Follow person	1257	2549		246	673		1011	1876	
**4 types of tumor** [a]									
IR (1/10^5 person-month)	4.73	18.93		8.68	13.24		4.11	20.53	
HR (95% CI)	1.00	3.85*	(1.37- 10.80)	1.00	2.06	(0.24- 17.74)	1.00	4.62*	(1.41- 15.09)
**CRC**									
IR	3.55	6.77		8.67	4.41		2.74	7.44	
HR (95% CI)	1.00	1.86	(0.53- 6.50)	1.00	0.64	(0.05- 8.11)	1.00	2.59	(0.58- 11.59)
**Lymphoma**									
IR	0.00	6.29		0.00	6.61		0.00	6.19	
HR (95% CI)	-	-		-	-		-	-	
**Liver cancer**									
IR	1.18	5.80		0.00	2.20		1.37	6.82	
HR (95% CI)	1.00	4.43	(0.57- 34.30)	-	-		1.00	4.25	(0.55- 33.10)
**Skin cancer**									
IR	2.36	0.97		17.24	0.00		0.00	1.24	
HR (95% CI)	1.00	0.44	(0.06- 3.18)	1.00	-		-	-	

The analysis settings were the same as Supplement Table 1

Supplement Figure 1. HR and 95% CIs for hospitalization, operation, hepatitis B and TB activation among CD patients with different medication.

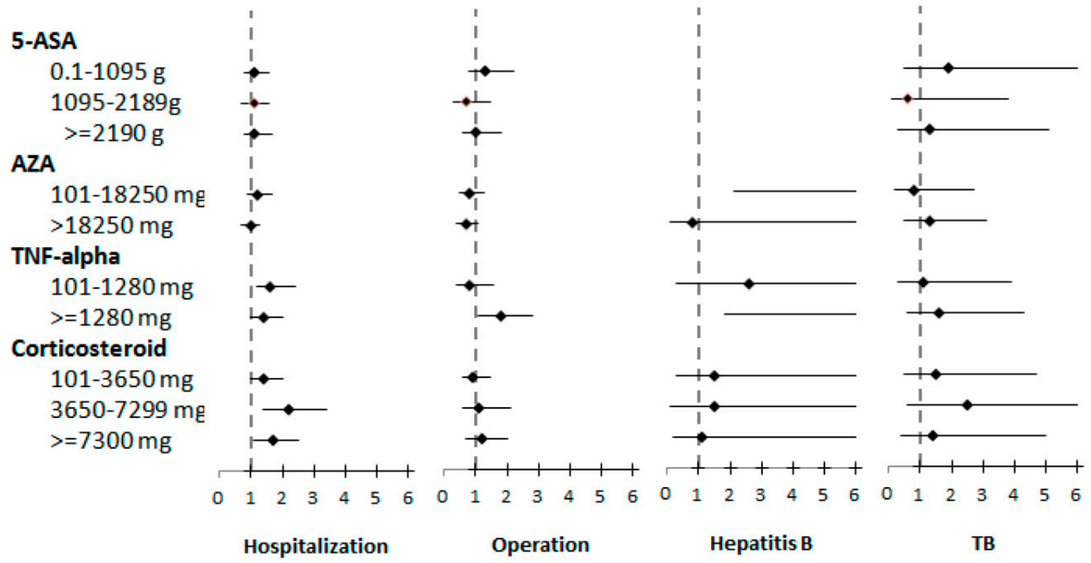

The analysis settings were the same as Figure 2.

Supplement Figure 2. HR and 95% CIs for hospitalization, operation, hepatitis B and TB activation among UC patients with different medication.

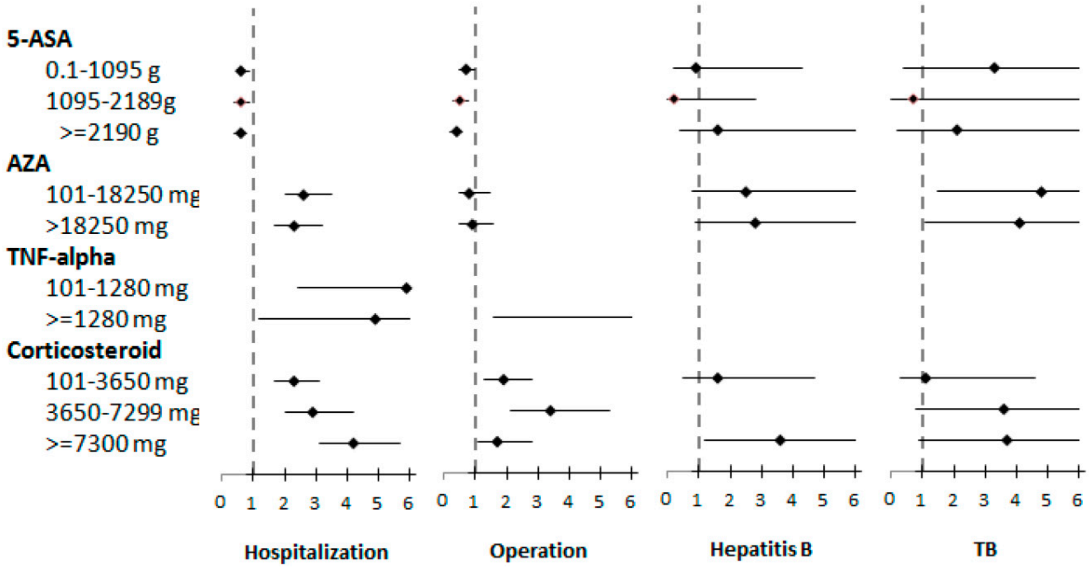

The analysis settings were the same as Figure 2.
